# Long-Term Effects of Sacubitril-Valsartan on Cardiac Remodeling: A Parallel Echocardiographic Study of Left and Right Heart Adaptive Response

**DOI:** 10.3390/jcm12072659

**Published:** 2023-04-03

**Authors:** Federica Valli, Francesca Bursi, Gloria Santangelo, Filippo Toriello, Andrea Faggiano, Irene Rusconi, Anna Maria Vella, Stefano Carugo, Marco Guazzi

**Affiliations:** 1 Department of Cardio-Thoracic-Vascular Diseases, Foundation IRCCS Cà Granda Ospedale Maggiore Policlinico, 20122 Milan, Italy; 2 Cardiology Division, San Paolo Hospital, 20142 Milan, Italy; 3 Department of Clinical Sciences and Community Health, University of Milan, 20122 Milan, Italy; 4 Montreal University Hospital Centre, Montreal, QC H2X 3E4, Canada

**Keywords:** heart failure with reduced ejection fraction, Sacubitril/Valsartan, global adaptive response, right heart function

## Abstract

Sacubitril/Valsartan (S/V) carries potential anti-remodeling properties, however long-term effects and biventricular adaptive response are poorly described. 76 HFrEF patients who underwent progressive uptitration of S/V, completed the annual scheduled follow-up. After a median follow-up of 11 (8–13) months, left ventricular (LV) reverse remodeling (RR) is defined as (1) absolute increase in LV ejection fraction (EF) ≥ 10% or LVEF ≥ 50% at follow-up and (2) decrease in indexed LV end-diastolic diameter (LVEDDi) of at least 10% or indexed LVEDDi ≤ 33 mm/m^2^, occurred in 27.6%. Non-ischemic etiology, shorter duration of HF, and absence of a history of AF were independently associated with LVRR (*p* < 0.05). TAPSE and TAPSE/PASP, a non-invasive index of right ventricular (RV) coupling to the pulmonary circulation, significantly improved at follow-up (0.45 vs. 0.56, *p* = 0.02). 41% of patients with baseline RV dysfunction obtained favorable RV remodeling despite only a moderate correlation between RV and LV function was observed (r = 0.478, *p* = 0.002). Our data point to a potential long-term reverse global remodeling effect by S/V, especially in patients who start S/V at an early stage of the disease, and focus our attention on a possible direct effect of the drug in synergistic hemodynamics between RV and pulmonary circulation.

## 1. Introduction

Sacubitril/Valsartan (S/V) is a Class I level of evidence B or A pharmacological approach to chronic heart failure with reduced ejection fraction (HFrEF) according to the European Society of Cardiology (ESC) [1] and the American Heart Association (AHA) [2]. In recent years many mechanisms of action have been proposed to explain the remarkable effects of S/V on long-term outcomes. Especially, findings by Zile et al. [3] obtained in a large number of samples, have pointed to the S/V effect on biomarkers of myocardial profibrotic signaling which are typically elevated in HFrEF. This evidence has driven attention to the potential anti-remodeling properties of S/V. In support of these findings, the PROVE-HF trial [4] and some real-life studies [5,6] have shown that S/V provides a significant effect on LV volumes and LV ejection fraction (EF). Moreover, the PRIME trial documented a significant modulation of secondary MR after 12 months of S/V treatment [7].

It is now established that Right ventricular (RV) remodeling and failure are the main determinants of the prognosis in patients with HFrEF [8,9] and that improvement of RV function is related to better survival [10]. Although pharmacodynamics of S/V, i.e., stimulation of cyclic guanosine monophosphate-protein kinase G pathway [11], is highly suggestive of a role of S/V in promoting an RV reverse remodeling, there is no long-term evidence but just some isolated reports in systemic RV [12] and HF (Heart Failure) [13].

The purpose of our study was to focus on the long-term effects of S/V on left and right remodeling by evaluating changes over time of conventional and emerging echocardiographic parameters, supporting the hypothesis that a parallel modulating activity on chamber geometry and function may explain the remarkable impact on hard end-points.

## 2. Materials and Methods

### 2.1. Clinical and Laboratory Data

This is a prospective observational study of HFrEF patients enrolled at the Heart Failure (HF) Clinic of our institute (January 2017–January 2021) who underwent a progressive uptitration to maximal doses of S/V. A Comprehensive Transthoracic Echocardiographic Examination, Clinical and laboratory data (creatinine, potassium, N-terminal pro-B-type natriuretic peptide -NT-proBNP) were collected prior to starting the treatment with S/V and at one year of follow-up. All data were collected on an electronic database (Microsoft Excel, Microsoft Office). S/V was started by the treating physician based on clinical indications and in keeping with recommendations that were available at the time of the study enrollment. According to the 2016 ESC guidelines for the diagnosis and treatment of acute and chronic HF, patients had to meet the following criteria to be included in the study: New York Heart Association (NYHA) class II–III; therapy with an individual optimal dose of beta blocker, angiotensin-converting-enzyme inhibitors (ACEi) or angiotensin receptor blockers (ARB); an echocardiographic examination documenting LVEF < 35% [14]. The exclusion criteria for the study were as follows: symptomatic hypotension and/or SBP < 100 mmHg; end-stage renal disease or estimated GFR < 30 mL/min/1.73 m^2^ (MDRD formula); serum potassium > 5.4 mmol/L at randomization; known history of hereditary or idiopathic angioedema or angioedema related to previous ACEI or ARB therapy; severe hepatic impairment. Per good clinical practice, after verifying the criteria for S/V initiation and the absence of contraindications, patients were prescribed S/V at a low or intermediate dose depending on systolic blood pressure and previous treatment with ACEi/ARB or naïve to drugs. Patients receiving ACEi therapy went through a 36 h washout period before starting administration. Doses of S/V were optimized to individual tolerance. Changes in doses of other medications were allowed when appropriate. Clinical data included age, sex, height, weight, body surface area (BSA), body mass index (BMI), and NYHA functional class, which were obtained by reviewing each patient’s medical charts. Comorbidities were documented by review of medical records and included hypertension (patients receiving antihypertensive medications or having blood pressure >140/90 mmHg), diabetes (patients receiving oral hypoglycemic or insulin medications), chronic obstructive pulmonary disease (COPD), chronic kidney disease (estimated glomerular filtration rate < 60 mL/min/1.73 m^2^—using The Modification of Diet in Renal Disease equation [15]) and a known history of atrial fibrillation -AF (paroxysmal, persistent or permanent). Patients were classified as having HFrEF of ischemic etiology based on a history of myocardial infarction or prior coronary percutaneous/surgical revascularization that could explain a remarkable cardiac dysfunction. The study protocol conforms to the ethical guidelines of the Declaration of Helsinki as reflected in a priori approval by the institution’s human research committee and was approved by the Clinical Research Ethics Committee of our institution. At the time of their baseline visit, all patients included agreed to be part of this study and signed written informed consent.

### 2.2. Echocardiographic Data

Transthoracic echocardiographic examinations were performed within a routine practice by trained sonographers and reviewed by experienced cardiologists with commercially available ultrasound equipment. Recordings were saved on a digital medium and post-processed on a workstation (Medimatic, ComPACS 10.10.22). Considering the observational design of our study, the timing of the follow-up echocardiograms ranged from 5 to 22 months. The echocardiographic data were retrieved unaltered from the original reports and inserted into the electronic database. The 2D echocardiographic linear measurement of LV internal diameter at end-diastole was obtained from the parasternal long axis view (LVEDD). LV end-diastolic volume (LVEDV) and LV end-systolic volume (LVESV) were measured from the apical two- and four-chamber views using the biplane modified Simpson’s rule; LVEDDi, LVEDVi, and LVESVi were obtained after adjusting for BSA using the Du Bois and Du Bois formula [16]. LVEF was calculated as (LVEDVi-LVESVi)/LVEDVi × 100. Left atrial volume was assessed using the modified biplane Simpson method from apical four-chamber and two-chamber views and indexed to BSA (LAVi). Measurements were obtained in end-systole from the frame preceding the mitral valve opening [17]. Diastolic function was evaluated according to international guidelines [18]. Mitral regurgitation (MR) severity was qualitatively graded as mild, mild to moderate, moderate, and severe. Pulmonary artery systolic pressures (PAsP) were estimated by calculating the systolic pressure gradient between the right ventricle and right atrium by the maximum velocity of the tricuspid regurgitant jet using the modified Bernoulli equation and then adding to this value the estimated right atrial pressures based on both the size of the inferior vena cava and the change in caliber of this vessel with respiration, according to the international recommendation. RV tricuspid annular plane systolic excursion (TAPSE), TAPSE/PASP ratio, and Fractional Area Change (FAC) were used to assess RV (RV) performance. Left ventricular reverse remodeling (LVRR) was defined as: (1) an absolute increase in LVEF ≥ 10 points or an LVEF ≥ 50% at follow-up and (2) a decrease in indexed LVEDD of at least 10% or an indexed LVEDD ≤ 33 mm/m^2^ [19]. In our study, patients with a TAPSE value lower than 17 mm at baseline and a TAPSE value greater than or equal to 17 mm at follow-up were considered to have obtained favorable RV remodeling.

### 2.3. Statistical Analysis

Continuous variables are presented as mean and standard deviation (SD) for normally distributed variables or as median and 25–75th percentile for asymmetrically distributed data, as appropriate. Categorical variables are summarized as frequencies and percentages. Pearson chi-squared test was used for comparison among categorical variables and Mann-Whitney U-test was used for continuous variables to test differences among independent samples. To examine changes in clinical and echocardiographic parameters before and after therapy with S/V the Wilcoxon Signed Rank test was used for continuous non-normally distributed data or ordinal variables and the McNemar test was used for categorical variables. The following formula was utilized to calculate the percentage change of echocardiographic parameters (i.e., ∆LVEF, ∆TAPSE) from baseline to follow-up: (follow-up parameter—baseline parameter)/baseline parameter ×100. Spearman correlation coefficient (r) was used to measure the strength of association between the left and RV functions. To examine the predictive value of baseline variables on LVRR, first, we performed a univariate analysis of all clinical-laboratory and echocardiographic parameters collected at enrollment; then, multivariable analysis was applied to the parameters that resulted significantly in the univariate analysis. SPSS for Windows version 21 (IBM Chicago, Chicago, IL, USA) was used for all the statistical analysis. Statistical significance was defined as two-tailed *p* < 0.05.

## 3. Results

### 3.1. Study Population

During the study period, 104 patients met the inclusion criteria. Among them, 18 patients were excluded due to a follow-up shorter than 5 months or because they were lost at follow-up. The final study population consisted of 86 patients. The baseline characteristics of the study population are shown in Table 1.

The mean age was 69 ± 12 years, 78% were males, and 49% had non-ischemic etiology. At the time of the start of S/V, the median duration of HFrEF since the first diagnosis was 28 (7–58) months. 35 (41%) patients had received cardiac resynchronization therapy (CRT) at a median of 20 months before starting S/V. Only 10 (12%) subjects were implanted with a CRT at the same time or slightly after S/V introduction. 64 (74%) patients had an implantable cardioverter defibrillator (ICD). Patients were on optimized medical therapy at the highest tolerated dose with 92% receiving ACEi/ARB, 93% beta-blockers, and 58% MRA.

#### Clinical and Echocardiographic Data at Baseline and Follow-Up

**Figure 1:**
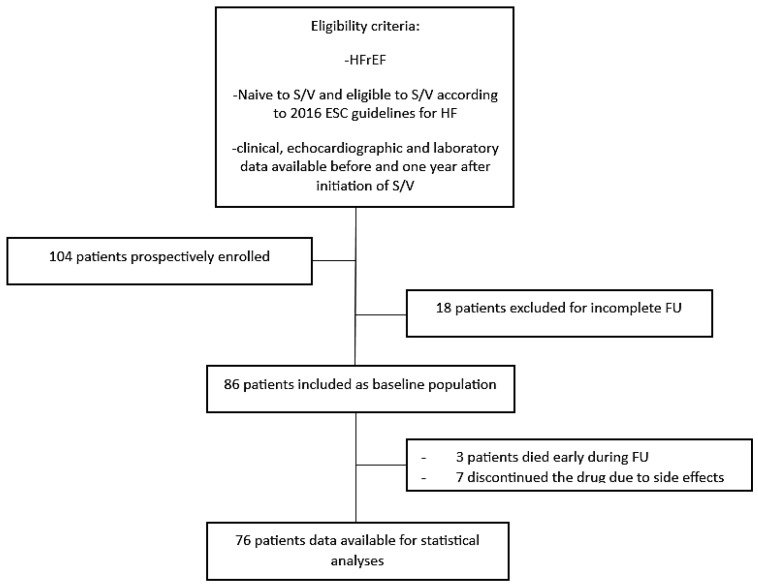
Study flow-chart.

The median duration of follow-up was 11 (25–75th percentile, 8–13) months since S/V was started. During follow-up 3 patients died before the scheduled follow-up echocardiogram, 7 discontinued the drug earlier than 12 months due to side effects (2 because of hypotension, 4 because of chronic kidney disease, 1 because of idiosyncratic reaction). Therefore, 76 patients had data available to compare follow-up versus baseline and specifically to evaluate heart remodeling (Figure 1). S/V was titrated to a maximally tolerated dose in each patient (median 200 mg; 100–400); 23 out of 76 patients (27%) were able to tolerate the highest dose.

As shown in Table 2, at baseline, left ventricular systolic function was severely reduced, and median EF was 30% (25–75th percentile 25–34%); LV dimensions and volumes were dilated both in diastole and systole. LVEF improved from 30% to 36% (*p* < 0.001) and LV diastolic and systolic volumes decreased significantly (*p* < 0.001). LAVi was enlarged at baseline and decreased significantly at follow-up along with the degree of mitral regurgitation and PASP. The median TAPSE significantly increased compared to baseline, and consequently, the median TAPSE/PASP ratio significantly improved at follow-up (*p* = 0.02). RAESVi also significantly decreased (*p* = 0.009). Among clinical and laboratory variables, NYHA class improved compared with baseline (*p* = 0.001) and NT-proBNP levels decreased significantly (*p* = 0.022), while renal function remained substantially unchanged.

### 3.2. Cardiac Reverse Remodeling

#### 3.2.1. Positive Left Ventricular Remodeling

LVRR occurred in 21 out of 76 patients (27.6%) (Table 3).

Patients with LVRR were younger, had a shorter duration of HFrEF, and had a non-ischemic etiology. Patients with no LVRR showed a greater prevalence of chronic renal disease and a history of AF. The daily dose of beta-blockers and MRA at baseline among patients with and without LVRR was comparable; the dose of S/V at follow-up was similar in the two groups. The distribution of CRT was similar in the two groups. Among echocardiographic parameters, LVEF significantly improved in both groups, but the change was more remarkable in patients with LVRR (*p* < 0.001). As expected at follow-up LVRR patients showed significant improvement in LVEDDi (*p* = 0.0004), LVEDVi (*p* = 0.002), LVESVi (*p* < 0.001, not shown in Table 3), and LAVi (*p* < 0.001). About functional RV parameters, TAPSE, PASP, and TAPSE/PASP ratio were at lower limits of the normal range in the two groups, at baseline. At follow-up, PASP improved in both groups (*p* = 0.068), TAPSE improved in both groups but more noticeably in LVRR patients (*p* < 0.005) along with TAPSE/PASP ratio (*p* = 0.004). At logistic regression analysis (Table 4), non-ischemic etiology, shorter duration of HF, and absence of a history of AF were independently and significantly associated with LVRR. Younger age was borderline significant *p* = 0.058.

#### 3.2.2. Favourable RV Remodeling

In a subgroup of patients, it was possible to examine the parameters of RV remodeling. In 15 patients TAPSE was not assessed at baseline, of the remainder 61 patients, 25 (41%), had a TAPSE lower than 17 mm at baseline.

Favorable RV remodeling was obtained in 41% of patients with baseline RV dysfunction at one year of follow-up.

At baseline, patients with and without RV remodeling had similar RV function and comparable loading conditions as shown by similar PASP (*p* for PASP at baseline = 0.549) and NT-proBNP values (*p* for NT-proBNP at baseline = 0.104).

TAPSE/PASP ratio was pathologically reduced at baseline in both groups and significantly improved at follow-up in the favorable remodeling group (0.73 vs. 0.43, *p* = 0.019); moreover, patients with RV function improvement, showed a better left ventricular function at follow-up (45% vs. 35%, *p* = 0.003).

Regarding interactions between heart structural changes and clinical disease status, patients with RV remodeling showed lower values of cardiac biomarkers at follow-up (NT-proBNP value 118 pg/mL vs. 1272 pg/mL, *p* = 0.03) and a trend for lower mortality rates (11% vs. 46%, *p* = 0.08).

25 out of 36 patients (70%) with normal TAPSE value at baseline maintained a normal RV function during SV treatment, and only 4 (10%) deteriorated.

In bivariate correlational analysis, ΔLVEF moderately correlated with ΔTAPSE (r = 0.478, *p* = 0.002), ∆ Fractional Area Change (FAC) (r = 0.430, *p* = 0.001), and ∆TAPSE/PASP (r = 0.669, *p* = 0.001). No correlations were found between ∆LVEF and ∆RVEDD (r = −0.012, *p* = 0.922).

## 4. Discussion

The present study shows that, in a population of patients with HFrEF, S/V uptitration to optimal dose, is associated with remarkable long-term reverse remodeling effect of both the left and the right heart. The parallel evaluation of LV and RV response over time represents a new approach rendering the concept of cardiac remodeling comprehensive, potentially implementing knowledge on the beneficial effects of S/V on the progressive history of RV enlargement and maladaptive response to pulmonary vascular load i.e., RV–pulmonary arterial uncoupling.

Pharmacological therapy remains the cornerstone for treating HFrEF. ACEi, ARB, beta-blockers, and MRAs have been shown to significantly reduce morbidity and mortality in this population and contribute to promoting beneficial anti-remodeling activity. However, despite this background medical therapy, the proportion of patients with persistent reduced systolic function remains high and portends an increased risk for heart failure hospitalization and cardiovascular death. The PARADIGM trial [20] showed that S/V significantly reduced both HF hospitalizations and cardiovascular mortality in comparison to guideline-recommended doses of enalapril. A number of studies have pointed to LV remodeling as one of the main determinants of this survival benefit [5,21,22,23], but very few data are available about the effects of S/V on RV performance [12,13].

In our study, LVEF was severely depressed at baseline and improved from 30% to 36% (*p* <0.001). This change is equivalent to the one observed in the PROVE-HF trial in which LVEF raised from 28.2% at baseline to 37.8% after 12 months [4]. In agreement with the results of Martens et al. [22] and the PROVE-HF study [4] we also found a significant reduction in left ventricular and atrial volumes and mitral regurgitation degree at follow-up.

We defined LVRR according to the definition by Merlo et al., to categorize it as improved only in patients in whom a benefit on major cardiac events had been demonstrated [19]. We observed the occurrence of LVRR in 21 out of 76 patients (27.6%), which is consistent with data reported by Sinagra et al. [5].

Considering the time the study was conducted, almost all patients had chronic HFrEF before the start of the drug, however, most of the HFrEF medical therapies remained substantially unchanged during the follow-up. Only 10 (12%) patients received a CRT at the same time or slightly after S/V introduction, but this proportion is unlikely to have affected the results.

In our study, although LVEF significantly improved in both patients with and without LVRR at follow-up, the change was more remarkable in patients with positive LVRR (45% and 33% respectively, *p* < 0.001). Furthermore, a significant decrease in left ventricular and atrial volumes was observed in the LVRR group. LAVI reflects the magnitude of elevated cardiac filling pressures. In patients with HFrEF, an increase in LA pressure and a reduction in LA compliance leads to LA remodeling resulting in an increase in LA stiffness, which may contribute to the development of pulmonary hypertension [24]. As LA volume and pressure increase, LA no longer acts as a barrier between the high left ventricular pressure and the pulmonary vessels [25], thus resulting in a passive transmission of the left ventricular pressure into the pulmonary vascular tree which determines isolated postcapillary pulmonary hypertension [26]. Younger age, a shorter duration of the disease, non-ischemic etiology, and sinus rhythm identified a subgroup of patients in whom LVRR is more likely to occur, confirming data from the literature. The beneficial effects of S/V were blunted in patients with a longer history of HFrEF (as shown by the longer duration of the disease in the no LVRR group); the relevant prognostic inhibition of profibrotic signaling promoted by S/V [3], allows us to speculate that the potentiality of S/V in preventing global cardiac remodeling could act in an early and reversible stage of the disease, supporting the assumption of PIONEER-HF trial [27] and reinforcing recent recommendations on HFrEF management [1].

Broadening the spectrum of cardiac remodeling to the right side, we observed that switching from ACEi/ARB to S/V impacted echocardiographic parameters of right heart function including TAPSE (*p* = 0.004) and PASP (*p* = 0.034) at follow-up. TAPSE/PASP ratio, a non-invasive index of RV coupling to the pulmonary arterial circulation (RV-PA coupling) also improved at follow-up (*p* = 0.020).

Already in the aforementioned Daunia registry [28] and in the complementary study of Armentaro et al. [29], 60 HFrEF outpatients followed for 6–12 and 24 months after the introduction of S/V obtained an improvement in NYHA class, NT-proBNP levels and in echocardiographic left and right heart parameters including TAPSE, PASP, right atrium area, and RV outflow tract diameter. To date, only Masarone et al. in 215 patients with HFrEF [13], reported improvement in RV-pulmonary arterial coupling through the measurement of TAPSE/PASP ratio after 2 years of treatment with S/V, which was independent of left ventricular remodeling parameters. Therefore, although there is still no large evidence in the literature, our data support and confirm findings of available observational studies reporting the benefits of S/V on right heart function [30].

The most novel finding of the present investigation is that the extent of S/V benefits were more evident on RV function. 41% of patients with RV dysfunction at baseline showed a positive remodeling after one year of treatment with S/V, with a more favorable ventricular to arterial coupling (*p* = 0.019). This effect appears to be independent of the severity of RV dysfunction at baseline and hemodynamic loading, conditions in this subgroup of patients. In HFrEF, a low TAPSE value (i.e., lower than 17 mm), a measure of RV systolic dysfunction, indicates an advanced disease stage and leads to an increased risk of death (both due to worsening HF and sudden death), and hospitalizations [31,32]. Conversely, RV recovery during follow-up is associated with improved survival in patients with chronic HFrEF [10]. Moreover, the TAPSE/PASP ratio, an emerging parameter that defines the adaptation of the RV to its afterload, is a strong determinant of functional capacity and survival in patients with HF [33,34] as well as in associated disease conditions [35]. The TAPSE/PASP ratio has been shown to be an independent predictor of disease severity and prognosis with a cutoff <0.36 mmHg/mm identifying patients with a high risk of cardiac events, irrespective of EF status [33]. In agreement with this knowledge, in our study patients with RV improvement showed lower levels of prognostic cardiac biomarkers at follow-up (NT-proBNP values 118 pg/mL vs. 1272 pg/mL, *p* = 0.03) and a trend for lower mortality rates (11% vs. 46%, *p* = 0.08).

LV function improvement obtained after S/V has a role in RV remodeling. The beneficial effects of S/V on biventricular structure and function may be partly explained by hemodynamic effects on peripheral resistance and natriuresis with a reduction of LV filling pressures and better LV-RV interactions [36,37]. However, the moderate correlation proved between right and ventricular function during follow-up in our analysis, lead us to consider several mechanisms linked to a beneficial effect of S/V on the RV side. As a matter of fact, pulmonary pressure overload in preclinical models clearly showed that S/V decreased pulmonary pressure and vascular remodeling, RV maximum pressures, improved RV contractile and relaxation functions, and prevented RV-PA uncoupling. After all, the beneficial effects of natriuretic peptides in the lung have been extensively described [38,39,40]. In our study, TAPSE, TAPSE/PASP, and FAC emerged as the best echocardiographic parameters to evaluate changes over time of right ventricular function; RVEDD emerged as a parameter with poor accuracy, possibly due to limitations of 2D echocardiography.

Moreover and not least, in our study S/V prevented the evolution of RV dysfunction also in patients with preserved TAPSE at enrolment.

## 5. Limitations

As in all observational studies our HFrEF patients were treated with other disease-modifying drugs which may have positively affected cardiac remodeling. However, considering the time the study was conducted, most patients had chronic HF before the start of the drug and in the majority, the HF therapies remained substantially unchanged during the study.

We acknowledge the small number of patients enrolled as the main limitation. Thus our results need to be confirmed in larger trials.

We also acknowledge that echocardiographic data and data on RV remodeling were not available in some patients.

## 6. Conclusions

Our data point to a potential long-term left and right reverse global remodeling effect by S/V, especially in patients who begin S/V at earlier stages. Findings recall attention on a possible direct effect of the drug in the coupling physiology between RV and pulmonary circulation. Overall, our data may support the hypothesis that the main benefits of S/V on hard end-points could be, at least in part, the result of these biventricular cardiac-related effects.

## Figures and Tables

**Table 1:** Characteristics of the study population at baseline. Continuous variables are presented as average and standard deviation or median and 25–75th percentile as appropriate. Categorical variables are presented as absolutes and percentages.

Baseline Characteristics	Total Population N = 86
Age (years)	69 ± 12
Male gender no. %	59 (78%)
BMI (kg/m^2^)	26 (23–30)
Duration of follow-up (months)	11 (8–13)
Time since the first diagnosis of HFrEF (months)	28 (7–58)
Non Ischemic etiology, no. %	42 (49%)
CAD, no. %	47 (55%)
SBP (mmHg)	130 (120–140)
DBP (mmHg)	80 (70–80)
HR (b/min)	69 (60–75)
NYHA Class, no. % II III	48 (56%) 35 (41%)
Hypertension, no. %	59 (69%)
History of AF, no. %	28 (32%)
Diabetes mellitus, no. %	24 (28%)
COPD, no. %	18 (21%)
Chronic kidney disease, no. %	24 (28%)
Creatinine (mg/dL)	1 (0.90–1.30)
EGFR (MDRD), (mL/min/1.73 m^2^)	76 (56–87)
Potassium (mmol/L)	4.3 (4.1–4.8)
NT-proBNP (ng/mL)	1520 (618–3002)
Beta-blockers, no. %	80 (93%)
ACEi/ARB, no. %	79 (92%)
MRA, no. %	50 (58%)
Diuretics, no. %	73 (85%)
Ivabradine, no. %	10 (12%)
Amiodarone, no. %	17 (20%)
ICD, no. %	64 (74%)
CRT, no. %	35 (41%)

BMI, Body Mass Index; CAD, Coronary Artery Disease; SBP, Systolic Blood Pressure; DBP, Diastolic Blood Pressure; HR, Heart Rate; NYHA, New York Heart Association; AF, Atrial Fibrillation; COPD, Chronic Obstructive Pulmonary Disease; EGFR, Estimated Glomerular Filtration Rate; NT-proBNP, B Type Natriuretic Peptide, ACEi/ARB Angiotensin-Converting-Enzyme Inhibitors/Angiotensin Receptor Blockers; MRA, Mineralocorticoid Receptor Antagonist; ICD, Implantable Cardioverter Defibrillator; CRT, Cardiac Resynchronization Therapy; HFrEF, heart failure with reduced ejection fraction; MDRD: Modification of Diet in Renal Disease.

**Table 2:** Comparison between baseline and follow-up clinical, laboratory, and echocardiographic parameters applying the Wilcoxon Signed Rank for continuous variables (paired samples) or the McNemar for categorical variables (paired samples).

Echocardiographic and Clinical Parameters (N = 76)	Baseline	Follow-Up (12 Months)	*p*-Value
LVEF (%)	30 (25–34)	36 (30–42)	<0.001
LVEDDi (mm/m^2^)	33 (30–36)	31 (28–35)	0.006
LVEDVi (mL/m^2^)	90 (74–107)	78 (58–92)	<0.001
LVESVi (mL/m^2^)	60 (49–81)	47 (32–64)	<0.001
LAVi (mL/m^2^)	62 (49–80)	47 (32–64)	<0.001
MR, no. % -moderate/severe -others (absent; mild)	20 (26) 56 (74)	10 (13) 66 (87)	0.013
RAESVi	30 (21–43)	26 (19–37)	0.009
RVEDD	38 (31–42)	34 (30–39)	0.062
TAPSE	18 (15–21)	19 (15–22)	0.004
PASP (mmHg)	37 (27–49)	30 (26–39)	0.034
TAPSE/PASP	0.45 (0.30–0.63)	0.56 (0.44–0.76)	0.020
NYHA class	2 (2–3)	2 (2–2)	0.001
SBP (mmHg)	130 (120–140)	120 (110–136)	0.018
HR (bpm)	67 (60–74)	67 (60–75)	0.497
Creatinine mg/dL	1 (0.89–1.28)	1.02 (0.89–1.34)	0.315
EGFR(MDRD) (mL/min/1.73 m^2^)	77 (56–87)	72 (53–86)	0.656
Potassium (mmol/L)	4.3 (4.2–4.8)	4.7 (4.2–4.9)	0.002
NT-proBNP (ng/mL)	1475 (567–3151)	1000 (418–2590)	0.022

LVEF, Left Ventricular Ejection Fraction; LVEDDi, Left Ventricular End-Diastolic Diameter indexed; LVEDVi, Left Ventricular End Diastolic Volume indexed; LVESVi, Left Ventricular End Systolic Volume indexed; LAVi, Left Atrial Volume indexed; MR, Mitral Regurgitation; RAESVi, Right Atrial End Systolic Volume indexed; RVEDD, RV End Diastolic Diameter; TAPSE, Tricuspid Annular Plane Systolic Excursion; PASP, Pulmonary Artery Systolic Pressures; SBP, Systolic Blood Pressure; HR, Heart Rate; NYHA, New York Heart Association; EGFR, Estimated Glomerular Filtration Rate; NT-proBNP, B Type Natriuretic Peptide.

**Table 3:** Comparison between clinical and echocardiographic parameters in patients with and without positive left ventricular cardiac remodeling.

	LVRR (N = 21)	No LVRR (N = 55)	*p* Value
Male sex, no. %	14 (67%)	45 (82%)	0.156
Age (years)	61 ± 12	71 ± 11	0.001
Time since the first diagnosis of HFrEF (months)	7 (2–28)	31 (11–58)	0.015
Duration of S/V (months)	11 (10–13)	11 (9–13)	0.963
Etiology, no. % -Ischemic -Non-ischemic	4 (19%) 17 (81%)	36 (65%) 19 (34%)	0.0003
History of AF, no. %	1 (5%)	19 (35%)	0.008
History of renal failure, no. %	2 (10%)	17 (31%)	0.054
Creatinine mg/dL	1 (0.8–1.1)	1 (0.9–1.3)	0.096
EGFR(MDRD), (mL/min/1.73 m^2^)	80 (73–88)	76 (56–87)	0.103
NYHA Class (I; II; III)	1		0.526
NT-proBNP ng/dL	893 (491–1735)	1750 (618–3313)	0.093
CRT, no. %	5 (24%)	27 (49%)	0.417
ICD, no. %	15 (71%)	39 (71%)	0.493
LVEF baseline % LVEF follow-up % LVEF > 35% at follow-up	30 (21–32) 45 (43–51) 20 (95%)	30 (25–35) 33 (28–38) 18 (33%)	0.141 <0.001 <0.001
LVEDDi baseline LVEDDi follow-up	33 (29–35) 29 (27–31)	33 (30–38) 33 (29–37)	0.630 0.0004
LVEDVi baseline LVEDVi follow-up	88 (75–104) 58 (48–77)	98 (71–117) 81 (64–98)	0.523 0.002
LAVi baseline LAVi follow-up	40 (37–46) 27 (22–38)	48 (42–59) 44 (36–54)	0.265 <0.001
RAESVi baseline RAESVi follow-up	21 (17–31) 21 (19–25)	32 (22–49) 30 (18–40)	0.05 0.067
RVEDD baseline RVEDD follow-up	34 (28–39) 33 (31–37)	38 (34–44) 35 (30–41)	0.048 0.285
TAPSE baseline TAPSE follow-up	18 (15–20) 21 (18–24)	18 (15–21) 17 (14–21)	0.949 0.004
PASP baseline PASP follow-up	36 (23–52) 27 (25–31)	37 (27–48) 32 (27–41)	0.587 0.068
TAPSE/PASP baseline TAPSE/PASP follow-up	0.50 (0.29–0.64) 0.73 (0.61–0.93)	0.45 (0.30–0.66) 0.48 (0.38–0.74)	0.939 0.004
Beta-blockers -none -Bisoprolol -Carvedilol -Metoprolol	1 (5%) 13 (65%) 5 (25%) 1 (5%)	2 (3.8 %) 31 (58.5%) 14 (26.4%) 6 (11.3%)	0.856
ACEI/ARB Ramipril Enalapril Valsartan Others	9 (43%) 2 (10%) 6 (29%) 3 (14%)	29 (53%) 7 (13%) 6 (11%) 9 (16%)	0.501
MRA at baseline (mg)	25 (25–34)	25 (25–25)	0.079
Cumulative incidence of HF rehospitalization and Death (all-cause)	4 (19%)	20 (36%)	0.146
Death (all-cause)	2 (9%)	11 (20%)	0.278

LVRR, Left Ventricular Reverse Remodeling, was defined as an increase in the LVEF ≥ 10 points (or LVEF ≥ 50%) associated with a decrease ≥10% in indexed left ventricular end-diastolic diameter (LVEDD) or LVEDD ≤ 33 mm/m^2^ at follow-up evaluation. NYHA, New York Heart Association, AF, Atrial Fibrillation; EGFR, Estimated Glomerular Filtration Rate; NT-proBNP, B Type Natriuretic Peptide; ICD, Implantable Cardioverter Defibrillator; CRT, Cardiac Resynchronization Therapy; MRA, Mineralocorticoid Receptor Antagonist; LVEF, Left Ventricular Ejection Fraction; LVEDDi, Left Ventricular End-Diastolic Diameter indexed; LVEDVi, Left Ventricular End Diastolic Volume indexed; LVESVi, Left Ventricular End Systolic Volume indexed; LAVi, Left Atrial Volume indexed; MR, Mitral Regurgitation; RAESVi, Right Atrial End Systolic Volume indexed; RVEDD, RV End Diastolic Diameter; TAPSE, Tricuspid Annular Plane Systolic Excursion; PASP, Pulmonary Artery Systolic Pressures.

**Table 4:** Logistic regression analysis, with LVRR as the dependent variable.

LVRR	OR	95% CI	*p* Value
Absence of history of AF	18.147	1.727–190.636	0.016
Non-ischemic etiology	12.839	2.941–56.052	0.001
Time since first diagnosis of HFrEF	0.977	0.954–0.999	0.043
Age	0.943	0.888–1.002	0.058

LVRR, Left ventricular reverse remodeling, AF, Atrial Fibrillation.

## Data Availability

The data presented in this study are available on request from the corresponding author.

## References

[1] McDonagh T.A., Metra M., Adamo M., Gardner R.S., Baumbach A., Böhm M., Burri H., Butler J., Čelutkienė J., Chioncel O. (2021). 2021 ESC Guidelines for the diagnosis and treatment of acute and chronic heart failure. Eur. Heart J..

[2] Heidenreich P.A., Bozkurt B., Aguilar D., Allen L.A., Byun J.J., Colvin M.M., Deswal A., Drazner M.H., Dunlay S.M., Evers L.R. (2022). 2022 AHA/ACC/HFSA Guideline for the Management of Heart Failure: A Report of the American College of Cardiology/American Heart Association Joint Committee on Clinical Practice Guidelines. Circulation.

[3] Zile M.R., O’Meara E., Claggett B., Prescott M.F., Solomon S.D., Swedberg K., Packer M., McMurray J.J.V., Shi V., Lefkowitz M. (2019). Effects of Sacubitril/Valsartan on Biomarkers of Extracellular Matrix Regulation in Patients with HFrEF. J. Am. Coll. Cardiol..

[4] Januzzi J.L., Prescott M.F., Butler J., Felker G.M., Maisel A.S., McCague K., Camacho A., Piña I.L., Rocha R.A., Shah A.M. (2019). Association of Change in N-Terminal Pro-B-Type Natriuretic Peptide Following Initiation of Sacubitril-Valsartan Treatment with Cardiac Structure and Function in Patients with Heart Failure with Reduced Ejection Fraction. JAMA.

[5] Castrichini M., Manca P., Nuzzi V., Barbati G., De Luca A., Korcova R., Stolfo D., Lenarda A.D., Merlo M., Sinagra G. (2020). Sacubitril/Valsartan Induces Global Cardiac Reverse Remodeling in Long-Lasting Heart Failure with Reduced Ejection Fraction: Standard and Advanced Echocardiographic Evidences. J. Clin. Med..

[6] Polito M.V., Silverio A., Rispoli A., Vitulano G., Auria F., De Angelis E., Loria F., Gigantino A., Bonadies D., Citro R. (2020). Clinical and echocardiographic benefit of Sacubitril/Valsartan in a real-world population with HF with reduced ejection fraction. Sci. Rep..

[7] Kang D.H., Park S.J., Shin S.H., Hong G.R., Lee S., Kim M.S., Yun S.C., Song J.M., Park S.W., Kim J.J. (2019). Angiotensin Receptor Neprilysin Inhibitor for Functional Mitral Regurgitation. Circulation.

[8] Guazzi M., Dixon D., Labate V., Beussink-Nelson L., Bandera F., Cuttica M.J., Shah S.J. (2017). RV Contractile Function and its Coupling to Pulmonary Circulation in Heart Failure with Preserved Ejection Fraction: Stratification of Clinical Phenotypes and Outcomes. JACC Cardiovasc. Imaging.

[9] De Groote P., Fertin M., Goéminne C., Petyt G., Peyrot S., Foucher-Hossein C., Mouquet F., Bauters C., Lamblin N. (2012). Right ventricular systolic function for risk stratification in patients with stable left ventricular systolic dysfunction: Comparison of radionuclide angiography to echoDoppler parameters. Eur. Heart J..

[10] Dini F.L., Carluccio E., Simioniuc A., Biagioli P., Reboldi G., Galeotti G.G., Raineri C., Gargani L., Scelsi L., Mandoli G.E. (2016). Right ventricular recovery during follow-up is associated with improved survival in patients with chronic heart failure with reduced ejection fraction. Eur. J. Heart Fail..

[11] Emdin M., Aimo A., Castiglione V., Vergaro G., Georgiopoulos G., Saccaro L.F., Lombardi C.M., Passino C., Cerbai E., Metra M. (2020). Targeting Cyclic Guanosine Monophosphate to Treat Heart Failure: JACC Review Topic of the Week. J. Am. Coll. Cardiol..

[12] Zandstra T.E., Nederend M., Jongbloed M.R.M., Kiès P., Vliegen H.W., Bouma B.J., Tops L.F., Schalij M.J., Egorova A.D. (2021). Sacubitril/Valsartan in the treatment of systemic right ventricular failure. Heart.

[13] Masarone D., Errigo V., Melillo E., Valente F., Gravino R., Verrengia M., Ammendola E., Vastarella R., Pacileo G. (2020). Effects of Sacubitril/Valsartan on the right ventricular arterial coupling in patients with heart failure with reduced ejection fraction. J. Clin. Med..

[14] Ponikowski P., Voors A.A., Anker S.D., Bueno H., Cleland J.G., Coats A.J., Falk V., González-Juanatey J.R., Harjola V.P., Jankowska E.A. (2016). 2016 ESC Guidelines for the diagnosis and treatment of acute and chronic heart failure: The Task Force for the diagnosis and treatment of acute and chronic heart failure of the European Society of Cardiology (ESC). Developed with the special contribution of the Heart Failure Association (HFA) of the ESC. Eur. J. Heart Fail..

[15] O’Meara E., Chong K.S., Gardner R.S., Jardine A.G., Neilly J.B., McDonagh T.A. (2006). The Modification of Diet in Renal Disease (MDRD) equations provide valid estimations of glomerular filtration rates in patients with advanced heart failure. Eur. J. Heart Fail..

[16] Shuter B., Aslani A. (2000). Body surface area: Du Bois and Du Bois revisited. Eur. J. Appl. Physiol..

[17] Lang R.M., Badano L.P., Mor-Avi V., Afilalo J., Armstrong A., Ernande L., Flachskampf F.A., Foster E., Goldstein S.A., Kuznetsova T. (2015). Recommendations for cardiac chamber quantification by echocardiography in adults: An update from the American Society of Echocardiography and the European Association of Cardiovascular Imaging. J. Am. Soc. Echocardiogr..

[18] Nagueh S.F., Smiseth O.A., Appleton C.P., Byrd B.F., Dokainish H., Edvardsen T., Flachskampf F.A., Gillebert T.C., Klein A.L., Lancellotti P. (2016). Recommendations for the Evaluation of Left Ventricular Diastolic Function by Echocardiography: An Update from the American Society of Echocardiography and the European Association of Cardiovascular Imaging. J. Am. Soc. Echocardiogr..

[19] Merlo M., Pyxaras S.A., Pinamonti B., Barbati G., Di Lenarda A., Sinagra G. (2011). Prevalence and prognostic significance of left ventricular reverse remodeling in dilated cardiomyopathy receiving tailored medical treatment. J. Am. Coll. Cardiol..

[20] McMurray J.J., Packer M., Desai A.S., Gong J., Lefkowitz M.P., Rizkala A.R., Rouleau J.L., Shi V.C., Solomon S.D., Swedberg K. (2014). Angiotensin-neprilysin inhibition versus enalapril in heart failure. N. Engl. J. Med..

[21] Santangelo G., Bursi F., Toriello F., Valli F., Tombolini E., Torta D., Bosotti L., Massironi L., Carugo S. (2019). Sacubitril/Valsartan improves medium-term reverse left ventricular remodeling: Why wait?. J. Cardiovasc. Med..

[22] Martens P., Beliën H., Dupont M., Vandervoort P., Mullens W. (2018). The reverse remodeling response to Sacubitril/Valsartan therapy in heart failure with reduced ejection fraction. Cardiovasc. Ther..

[23] Villani A., Ravaro S., Cerea P., Caravita S., Ciambellotti F., Branzi G., Munforti C., Parati G., Malfatto G. (2020). Do the remodeling effects of Sacubitril/Valsartan treatment depend upon heart failure duration?. J. Cardiovasc. Med..

[24] Charalampopoulos A., Lewis R., Hickey P., Durrington C., Elliot C., Condliffe R., Sabroe I., Kiely D.G. (2018). Pathophysiology and Diagnosis of Pulmonary Hypertension Due to Left Heart Disease. Front. Med..

[25] Borlaug B.A. (2014). The pathophysiology of heart failure with preserved ejection fraction. Nat. Rev. Cardiol..

[26] Guazzi M., Naeije R. (2017). Pulmonary Hypertension in Heart Failure: Pathophysiology, Pathobiology, and Emerging Clinical Perspectives. J. Am. Coll. Cardiol..

[27] Velazquez E.J., Morrow D.A., DeVore A.D., Duffy C.I., Ambrosy A.P., McCague K., Rocha R., Braunwald E., PIONEER-HF Investigators (2019). Angiotensin-Neprilysin Inhibition in Acute Decompensated Heart Failure. N. Engl. J. Med..

[28] Correale M., Mallardi A., Mazzeo P., Tricarico L., Diella C., Romano V., Ferraretti A., Leopizzi A., Merolla G., Di Biase M. (2020). Sacubitril/valsartan improves right ventricular function in a real-life population of patients with chronic heart failure: The Daunia Heart Failure Registry. Int. J. Cardiol. Heart Vasc..

[29] Armentaro G., D’Arrigo G., Magurno M., Toscani A.F., Condoleo V., Miceli S., Cassano V., Maio R., Arturi F., Tripepi G. (2021). Impact of Sacubitril/Valsartan on Clinical and Echocardiographic Parameters in Heart Failure Patients with Reduced Ejection Fraction: Data From a Real Life 2-year Follow-Up Study. Front. Pharmacol..

[30] Zhang J., Du L., Qin X., Guo X. (2022). Effect of Sacubitril/Valsartan on the Right Ventricular Function and Pulmonary Hypertension in Patients with Heart Failure with Reduced Ejection Fraction: A Systematic Review and Meta-Analysis of Observational Studies. J. Am. Heart Assoc..

[31] Ghio S., Recusani F., Klersy C., Sebastiani R., Laudisa M.L., Campana C., Gavazzi A., Tavazzi L. (2000). Prognostic usefulness of the tricuspid annular plane systolic excursion in patients with congestive heart failure secondary to idiopathic or ischemic dilated cardiomyopathy. Am. J. Cardiol..

[32] Damy T., Kallvikbacka-Bennett A., Goode K., Khaleva O., Lewinter C., Hobkirk J., Nikitin N.P., Dubois-Randé J.L., Hittinger L., Clark A.L. (2012). Prevalence of, associations with, and prognostic value of tricuspid annular plane systolic excursion (TAPSE) among out-patients referred for the evaluation of heart failure. J. Card. Fail..

[33] Guazzi M., Bandera F., Pelissero G., Castelvecchio S., Menicanti L., Ghio S., Temporelli P.L., Arena R. (2013). Tricuspid annular plane systolic excursion and pulmonary arterial systolic pressure relationship in heart failure: An index of right ventricular contractile function and prognosis. Am. J. Physiol. Hear Circ. Physiol..

[34] Teramoto K., Sengelov M., West E., Santos M., Nadruz W., Skali H., Shah A.M. (2020). Association of pulmonary hypertension and right ventricular function with exercise capacity in heart failure. ESC Heart Fail..

[35] Bursi F., Santangelo G., Barbieri A., Vella A.M., Toriello F., Valli F., Sansalone D., Carugo S., Guazzi M. (2022). Impact of Right Ventricular-Pulmonary Circulation Coupling on Mortality in SARS-CoV-2 Infection. J. Am. Heart Assoc..

[36] Merlo M., Gobbo M., Stolfo D., Losurdo P., Ramani F., Barbati G., Pivetta A., Di Lenarda A., Anzini M., Gigli M. (2016). The Prognostic Impact of the Evolution of RV Function in Idiopathic DCM. JACC Cardiovasc. Imaging.

[37] Bouali Y., Galli E., Paven E., Laurin C., Arnaud H., Oger E., Donal E. (2022). Impact of Sacubitril/Valsartan on systolic heart failure: Right heart location and clustering analysis. Adv. Clin. Exp. Med..

[38] Sharifi Kia D., Benza E., Bachman T.N., Tushak C., Kim K., Simon M.A. (2020). Angiotensin Receptor-Neprilysin Inhibition Attenuates Right Ventricular Remodeling in Pulmonary Hypertension. J. Am. Heart Assoc..

[39] Clements R.T., Vang A., Fernandez-Nicolas A., Kue N.R., Mancini T.J., Morrison A.R., Mallem K., McCullough D.J., Choudhary G. (2019). Treatment of Pulmonary Hypertension with Angiotensin II Receptor Blocker and Neprilysin Inhibitor Sacubitril/Valsartan. Circ. Heart Fail..

[40] Potter L.R., Abbey-Hosch S., Dickey D.M. (2006). Natriuretic peptides, their receptors, and cyclic guanosine monophosphate-dependent signaling functions. Endocr. Rev..

